# Correlation between Renal Artery Anatomy and Hypertension: A Retrospective Analysis of 3000 Patients

**DOI:** 10.1155/2021/9957361

**Published:** 2021-12-29

**Authors:** Jiayi Shen, Lingchun Lyu, Xiaoyan Wu, Jiansong Ji, Chunlai Zeng, Shan Li, Yanan Zhao, Jian Xu, Li Lin, Chenyin Lu, Wei Mao, Tiemin Wei

**Affiliations:** ^1^Lishui Hospital, Zhejiang University School of Medicine, Lishui, Zhejiang 323000, China; ^2^Lishui Cardiovascular Clinical Research Center, Lishui, Zhejiang 323000, China; ^3^Key Laboratory of Imaging Diagnosis and Minimally Invasive Intervention Research, Lishui, Zhejiang 323000, China; ^4^Lishui Cardiovascular & Cerebrovascular Diseases Control Centre, Lishui, Zhejiang 323000, China; ^5^Zhejiang Hospital of Traditional Chinese Medicine, Hangzhou 310003, China

## Abstract

**Objective:**

To assess the correlation between renal artery anatomy and blood pressure in Undiagnosed Hypertension and Diagnosed Hypertension.

**Methods:**

The renal artery CT scanning imaging data and laboratory data of 3000 inpatients and outpatients were collected retrospectively in 4 centers of China. Morphometric parameters were assessed using the quantitative vascular analysis (unit: mM).

**Results:**

687 cases (23.2%) had accessory renal arteries unilaterally, and 216 cases (7.3%) had bilateral accessory renal arteries, including left kidney 825 (27.9%) and right kidney 798 (27.0%). The presence of accessory renal arteries and renal artery branches was higher in the diagnosed hypertension group as compared with the undiagnosed hypertension group (MARB, *pp* < 0.001; ARA, *p* < 0.001; others, *p* < 0.001). Consequently, multivariate regression analysis showed that age (OR = 2.519 (95% CI: 0.990–6.411, *p* < 0.001)), dyslipidemia (OR = 1.187 (95% CI: 0.960–1.454, *p* = 0.007)), renal hilum Outside the main renal artery branch (MRAB) (OR = 2.069 (95% CI: 1.614–2.524, *p* = 0.002)), and accessory renal artery (ARA) (OR = 2.071 (95% CI: 1.614–2.634, *p* = 0.001)) were risk factors of hypertension. In addition, higher renin activity was associated with ARA patients (2.19 ± 2.91 vs. 1.75 ± 2.85, *p* < 0.001).

**Conclusions:**

When comparing renal arteries side by side, the anatomical length of the renal arteries is significantly different. In addition, the prevalence of accessory renal arteries and renal artery branches is higher in the hypertension group. The auxiliary renal artery and the main renal artery branch outside the renal portal are independent factors of hypertension. Renal sympathetic nerve activity is affected by renin activity and is related to the accessory renal artery.

## 1. Introduction

Hypertension is highly prevalent and is related to the morbidity and mortality of cardiovascular diseases. Hypertension remains the major risk factor for the most significant cardiovascular events such as stroke, myocardial infarction, and heart failure and one of the most prevalent chronic diseases [1]. The causes of hypertension are complicated, and kidney is an important cause of various complications of hypertension, including renal parenchymal lesions and renal vascular lesions. With increasing attention to renal vascular causes and hypertension goals, more detailed knowledge of renal artery anatomy is urgently needed [2–7]. The former is due to acute and chronic glomerulonephritis, diabetic nephropathy, chronic pyelonephritis, polycystic kidney and kidney transplantation, and other kidney diseases [8], whereas the latter is due to renal artery disease, leading to renal ischemia and activation of the renin-angiotensin-aldosterone system. Few reports have investigated the correlation between the normal diameter of renal arteries and the occurrence and development of hypertension [9]. Changes in the anatomy of the renal artery may affect blood pressure control and hypertension. A recent study has shown that there is a correlation between the presence of accessory renal arteries and hypertension [10]. Knowledge of renal artery anatomy appears to be crucial for the understanding of the profound pathophysiology of hypertension and the development of intravascular treatment options [11]. Previous studies have reported that accessory renal arteries are significantly more common in patients with drug-resistant hypertension [12], but not in essential hypertension. There are also many correlations between anatomical variation of renal arteries and renal innervation therapy (RDN). During the 6-month follow-up, the diameter of the renal artery after RDN was related to changes in systolic blood pressure (SBP). The change of SBP has nothing to do with the length of renal artery and the existence of ARA [13]. Renal artery anatomy has been studied in people with refractory hypertension, but it is still lacking in people with general hypertension. This study attempts to explore the relationship between the anatomical structure of the kidneys, accessory renal arteries, and hypertension in 3,000 patients who underwent enhanced CT scan.

## 2. Materials and Methods

### 2.1. Study Population

The study is a multicenter retrospective analysis. The renal artery CT scanning imaging data and laboratory data of 3000 inpatients and outpatients were collected retrospectively in 4 medical institutions in southwest China. Patients were selected between the ages of 45 and 75, whose electronic medical records without a history of urinary system surgery and aldosteronism. Due to lack of data and the exclusion criteria, 46 patients were excluded, and 2954 patients were selected for retrospective analysis. Diagnosed hypertension is defined as systolic office blood pressure (SBP) ≥140 mmHg or diastolic pressure (DBP) of ≥90 mmHg or has received antihypertensive therapy. Undiagnosed hypertension has no history of hypertension or normal blood pressure. Automated office blood pressures (AOBP) were obtained through an automated oscillatory measuring device (e.g., Omron HEM-705 monitor, Omron Healthcare, Vernon Hills, Illinois, USA). All blood pressure measurements were performed in accordance with the guidelines of the VII Joint National Committee. Current antihypertensive drugs had been confirmed by electronic medical records.

### 2.2. Renal CT Scan

Siemens dual-source computed tomography (CT) scanner (Siemens Definitive, Siemens Medical, Forchheim, Germany) was used for scanning with iodine contrast agent (iodixanol, 320 mg I/ml) at a flow rate of 3.5–4.5 ml/*s*; the scan parameters were as follows: collimator 192 × 0.6 mm, rotation time 0.25 seconds, spacing 3.2, start CARE KV technology (tube voltage adjustment range 70–120 kV), automatic tube current adjustment technology (CARE Dose 4D), refer to 320 mAs/rot in milliseconds. The image postprocessing parameters were as follows: the thickness of the abdominal aortic reconstruction layer is 0.75 mm, the interval between the layers is 0.5 mm, and the convolution kernel is Bv40. The reconstruction of aorta ranged from thoracic spine to pubic symphysis, the thickness of reconstructed layer was 1.0 mm, the interval between layers was 0.7 mm, and the circumflex nucleus was Bv40. All raw data were transmitted to the DECT postprocessing workstation (Syngo version VB10, Siemens Healthcare, Forchheim, Germany). including volume rendering, curved multiplane reforming, and maximum intensity projection.

### 2.3. Renal Artery Quantitative Analysis

**Figure 1:**
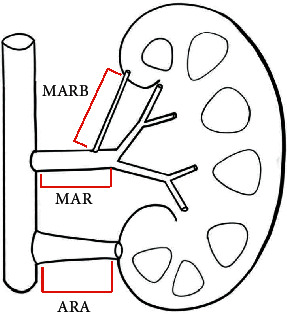
Naming rules of renal artery. Main renal artery branches outside the renal hilum (MRAB), main renal artery branches in the renal hilum (MRA) and the accessory renal artery branch of the abdominal aorta (ARA).

Morphometric parameters such as minimum, mean, and maximum diameter as well as length were assessed using the Discover syngo.via (Syngo VIA10B) as previously described (unit: mm) [13]. The accessory renal artery (ARA) was defined as a branch of abdominal aortic. MARB was defined as the main renal artery branch outside the renal hilum. Hemodynamically significant stenosis was excluded. The nomenclature of the renal arteries is shown in Figure 1. The parameter was recorded, and two experienced investigators were blind to the patient's characteristics.

### 2.4. Renin Activity Test

The data of renin activity were obtained in the electronic laboratory system. Laboratory measurement process: for patients who were not taking antihypertensive drugs, venous blood should be drawn from the lying position after lying down for two hours. If patients had taken antihypertensive drugs, they should stop taking antihypertensive drugs the day before the examination. The venous blood was quickly injected into a special anticoagulation tube, shaken immediately, placed in an ice water bath to cool, and then taken out during centrifugation. After centrifugation at 3000 rpm for 5 minutes, plasma was separated and renin levels were measured using a Maglumi 2000 automatic analyzer (SNIBE, Shenzhen, China).

### 2.5. Statistical Analysis

Data management and all statistical analyses were performed using IBM SPSS Statistics (version 23.0; SPSS Inc., Chicago, Illinois, USA). Data were presented as mean ± standard deviation (SD) for continuous variables and as numbers (%) for categorical variables unless otherwise stated. The comparison between two groups was performed using Pearson's *χ*^2^-test or Fisher's exact test for categorical variables and the Wilcoxon rank-sum test or the Kruskal–Wallis test for continuous variables (if applicable). *p* < 0.01 was considered statistically significant.

## 3. Results

### 3.1. Patients' Population

The average age of patients was 62.21 ± 11.38 years old, and that of men was 46.86%. The undiagnosed hypertension group and the diagnosed hypertension group included 2028 patients and 926 patients, respectively. Type 2 diabetes and coronary artery disease (CAD) were prevalent in 291 (9.85%) and 221 (7.48%) patients, respectively. There were statistically significant differences between the two groups in age (61.08 ± 11.67 vs. 63.79 ± 11.27), gender (male, 48.49% vs. 43.3%), drinkers (58.88% vs. 52.38%), GFR (69.74 ± 1.86 vs. 74.80 ± 2.36), dyslipidemia (15.64 vs. 28.19%), prevalence of type 2 diabetes (7% vs. 16.09%), and prevalence of CAD (5.42% vs. 11.99%) (Table 1; *p* < 0.01, all).

### 3.2. Comparison of Blood Pressure in Each Group

Of the 926 patients with hypertension, 799 (86.3%) had a history of antihypertensive medication. There were significant statistical differences in SBP (120.5 ± 10.9 mmHg vs. 140.9 ± 15.1 mmHg), DBP (70.0 ± 6.9 mmHg vs. 75.4 ± 8.6 mmHg), and pulse pressure (49.7 ± 9.6 mmHg vs. 63.7 ± 14.2 mmHg) of each group (Table 2; *p* < 0.01, all).

### 3.3. Comparative Renal Artery Anatomy

In all patients, the right main renal artery was longer than the left main renal artery (41.1 ± 12.4 vs. 33.8 ± 12.5 mm), whereas the right main renal artery was of slightly greater diameter (5.2 ± 1.1 vs. 5.4 ± 1.1 mm). The mean diameters of the main renal arteries were similar in patients with diagnosed hypertension than undiagnosed hypertension both in left or right main renal arteries (5.1 ± 1.1 vs. 5.3 ± 1.2 mm, 5.1 ± 1.3 vs. 5.2 ± 1.0 mm).

**Figure 2:**
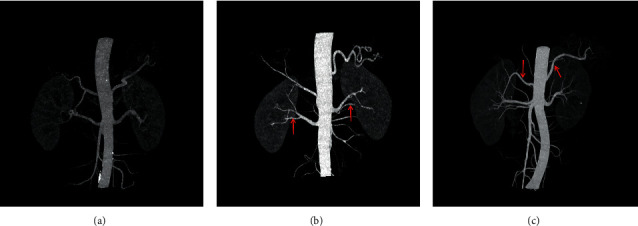
Types of renal artery and accessory renal artery described in enhanced renal CT scan. Different accessory renal arteries with renal CT scan. (a) No accessory renal artery (NAR); (b) main renal artery branches outside the renal hilum (MRAB); (c) the accessory renal artery (ARA).

No accessory renal artery (NAR) (69.4%), abdominal aortic (ARA) (15.6%), and main renal artery branches outside the renal hilum (MRAB) (14.6%) are shown in renal artery CT images (Figure 2, Table 3). Among these patients, there were 687 cases (23.2%) with unilateral accessory renal arteries, 216 cases (7.3%) with bilateral accessory renal arteries, 825 cases with left kidney (27.9%) and 798 cases with right kidney (27.0%). The presence of ARA and MARB was higher in the diagnosed hypertension group compared to the undiagnosed hypertension group (17.1% vs. 14.8%, 15.3% vs. 14.3%, both *p* < 0.001) (Table 3).

### 3.4. Analysis of Renal Artery Structure and Renin Activity

**Figure 3:**
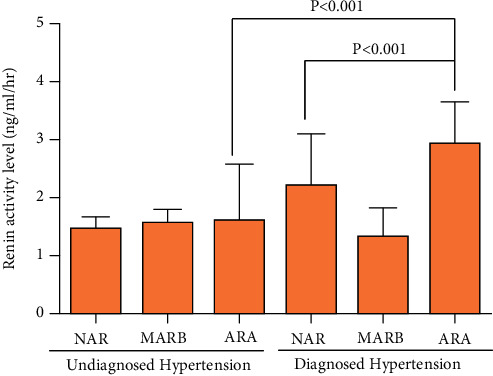
Renin activity with different types of renal artery and accessory renal artery. Quantitative analysis of renin activity in different subgroups. No accessory renal artery (NAR); main renal artery branches outside the renal hilum (MRAB), the accessory renal artery (ARA).

The higher renin activity was associated with ARA especially in the diagnosed hypertension group (Figure 3). The renin activity in patients with ARA of the diagnosed hypertension group (2.19 ± 2.91) was higher compared to patients with ARA of undiagnosed hypertension group (1.43 ± 2.07) and also higher than patients with NAR of diagnosed hypertension group (1.75 ± 2.85) (*p* < 0.001, both).

**Figure 4:**
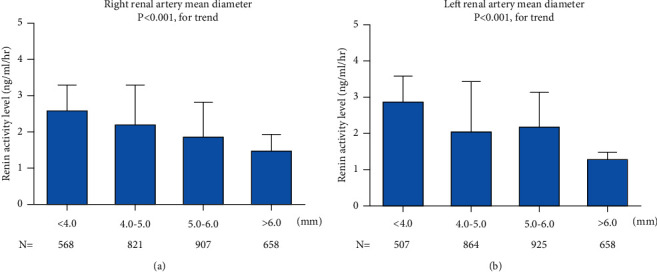
Renin activity of different renal artery mean diameter. Changes of renin activity according to the diameters of left (a) and right (b) renal artery and according to renal artery mean diameter. *p* values are ANOVA for trends.

However, on both sides, patients with a diameter ≤4.0 mm had higher levels of renin activity than patients with a diameter >4 mm (Figure 4). In ANOVA analysis, renin activity was statistically different in different renal artery diameters on the left (*p* < 0.001) and right (*p* < 0.001).

### 3.5. Adjusted Risks of the Accessory Renal Artery in Hypertension Patients

Univariate logistic regression and multivariate logistic regression analysis was performed to evaluate the correlation between hypertension and ARA in the diagnosed hypertension group (Tables 4 and 5). In model 1, the odds of ARA (OR = 0.954; 95% CI = 0.741–1.229; *p* = 0.718) for hypertension was 0.954. However, in model 2 that included adjustments for age, sex, drinker, smoker, BMI, dyslipidemia, and right renal artery mean diameter + left renal artery mean, regression analysis indicated that the ARA (OR = 2.071; 95% CI = 1.614–2.634; *p* = 0.001) was independent of the hypertension. It shows that the diameter of renal artery is very important to the correlation between ARA and hypertension.

## 4. Discussion

The present study analyzed the renal vascular structure and accessory renal artery of 3000 patients and subsequently introduced the relationship between accessory renal artery and hypertension. The renal artery originates outside the aorta below the superior mesenteric artery. The right renal artery is longer than the left renal artery because it passes through the inferior vena cava [14, 15]. Our findings also confirm this finding. We also compared the diameters of the left and right aortic arteries. The diameter of the left aortic artery is larger, but there is no statistical difference. However, Palmieri et al. found there is no difference between the diameter of the main renal arteries with and without accessory renal arteries [14]. But we did not find a correlation between the diameter and length of renal artery and hypertension.

The prevalence of accessory renal arteries in Chinese is 30.5% (23.2% unilaterally and 7.3% bilaterally) similar to previous studies [16, 17]. The prevalence of MRAB and ARA was higher in the diagnosed hypertension group compared to undiagnosed hypertension group, which is ranging from 14.3% to 15.3% and 14.8% to 17.1%, respectively. Previous studies have shown uncertainty about gender differences in prevalence [14, 16], and our records indicate that male renal artery is more common than female.

Our results indicate that there is a correlation between renal sympathetic nerve activity and the average diameter of accessory renal artery and renal artery. Increased length of accessory renal artery and plaque can cause hemodynamic changes [18, 19]. Therefore, due to pathophysiological mechanisms, differences in renal vascular structure may lead to changes in the renin-angiotensin-aldosterone system. A moderate decrease in renal blood flow may cause tissue hypoxia or damage, which may result in increased renal sympathetic nerve activity (RSNA). Consistent with this, our results indicate that the proportion of accessory renal arteries in patients with hypertension is higher (14.8% vs. 17.1%). The adjusted risk of accessory renal artery in hypertensive patients is 2.069 (*p* = 0.002). In the current multicenter retrospective analysis of the Chinese population, ARA is an independent risk factor for hypertension. Previous studies on hypertension in ARA [16, 18–20] has shown that ARA causes hypertension through the renin-angiotensin system. Angiographic study [18] has shown that the renal parenchyma slower blood flow and lower blood pressure leading to the perfusion defect in the supply area, thus resulting in increased renin secretion and increased blood pressure. A recent study also showed [19] that ARA was thinner and longer than the main renal artery, with lower perfusion pressure and higher blood flow resistance. Our results also confirm the link between renin and ARA and the diameter of the renal artery. It is suggested that lack of local perfusion may lead to increased renin secretion due to insufficient perfusion of renal artery and renal parenchyma [21, 22]. However, the general role of ARA in the development of hypertension is still elusive [23].

As with all studies, we should acknowledge the limitations of our work. Even though our CT scan shows the maximum diameter, minimum diameter, length, and type of accessory renal artery in the renal artery, we did not discuss differences in other geometric factors, such as angle effects. In addition, this study is a retrospective analysis of the structural differences between the accessory renal artery and renal aorta in patients diagnosed with hypertension, but no prospective analysis of the impact of these factors on hypertension. However, this study confirmed the correlation between renin secretion and accessory renal artery diameter and renal artery diameter, providing a new way to study the relationship between accessory renal artery and hypertension.

## 5. Conclusion

In conclusion, when comparing renal arteries by side, the anatomic length of the renal arteries is significantly different, but there is no difference in diameter. Furthermore, the prevalence of the accessory renal artery was higher in the hypertensive group in each type. It is worth noting that ARA was an independent factor of hypertension. Renal sympathetic nerve activity is affected by renin activity and is related to ARA and mean renal artery diameter.

## Figures and Tables

**Table 1:** Baseline data between undiagnosed hypertension and diagnosed hypertension.

	All patients	Undiagnosed hypertension	Diagnosed hypertension	*p* value
Number of patients	2954	2028	926	—
Age, years	62.21 ± 11.38	61.08 ± 11.67	63.79 ± 11.27	<0.01
Sex, male *n* (%)	1385 (46.86)	984 (48.49)	401 (43.30)	<0.01
Smoker, *n* (%)	1059 (35.86)	751 (37.03)	308 (33.30)	0.05
Drinker, *n* (%)	1678 (56.84)	1193 (58.88)	485 (52.38)	<0.001
GFR, ml/(min ^*∗*^ 1.73 m^2^)	72.69 ± 1.92	69.74 ± 1.86	74.80 ± 2.36	<0.01
Dyslipidemia	578 (19.57)	317 (15.64)	261 (28.19)	<0.001
Type 2 diabetes, *n* (%)	291 (9.85)	142 (7.00)	149 (16.09)	<0.001
CAD, *n* (%)	221 (7.48)	110 (5.42)	111 (11.99)	<0.001

GFR = glomerular filtration rate; standard of dyslipidemia: triglyceride >1.7 mmol/l or total cholesterol >5.17 mmol/l; CAD, coronary artery disease.

**Table 2:** Comparison of blood pressures, diagnostic rate of hypertension, and pulse pressure in each group.

	All patients	Undiagnosed hypertension	Diagnosed hypertension	*P* value
Number of patients	2954	2028	926	—
Systolic blood pressure (mmHg)	129.5 ± 16.4	120.5 ± 10.9	140.9 ± 15.1	<0.001
Diastolic blood pressure (mmHg)	72.4 ± 8.1	70.0 ± 6.9	75.4 ± 8.6	<0.001
Pulse pressure (mmHg)	55.9 ± 13.7	49.7 ± 9.6	63.7 ± 14.2	<0.001
Number of antihypertensive dugs, *n* (%)	799 (27.0)	—	799 (86.3)	—

**Table 3:** Comparison between undiagnosed hypertension and diagnosed hypertension.

	All patients	Undiagnosed hypertension	Diagnosed hypertension	*p* value
Left main renal artery (LMA)				
Length, mm	33.8 ± 12.5	32.5 ± 11.8	34.8 ± 12.5	0.153
Minimum diameter, mm	4.4 ± 1.1	4.8 ± 1.6	4.2 ± 1.0	0.624
Mean diameter, mm	5.4 ± 1.1	5.3 ± 1.2	5.1 ± 1.1	0.765
Maximum diameter, mm	8.2 ± 1.7	8.1 ± 1.6	8.3 ± 1.5	0.394

Right main renal artery (RMA)				
Length, mm	41.1 ± 12.4	41.3 ± 12.1	40.2 ± 11.5	0.287
Minimum diameter, mm	4.3 ± 1.2	4.2 ± 1.31	4.3 ± 1.4	0.167
Mean diameter, mm	5.2 ± 1.1	5.2 ± 1.0	5.1 ± 1.3	0.124
Maximum diameter, mm	6.8 ± 1.6	6.6 ± 1.5	6.9 ± 1.7	0.827

Accessory renal artery				
Left kidney, *n* (%)	825(27.9)	521(25.6)	304(32.8)	<0.001
Right kidney, *n* (%)	798(27.0)	491(24.2)	307(33.1)	<0.001
MARB, *n* (%)	433(14.6)	291(14.3)	132(15.3)	<0.001
ARA, *n* (%)	460(15.6)	301(14.8)	159(17.1)	<0.001
Others, *n* (%)	10(0.4)	4(0.2)	6(0.6)	<0.001
Unilateral, *n* (%)	687(23.2)	486(23.9)	201(21.7)	0.178
Bilateral, *n* (%)	216(7.3)	155(7.6)	61(6.5)	0.307

LMA = left main renal artery; RMA = right main renal artery. Others, other branches of renal artery.

**Table 4:** Univariate logistic regression for diagnosed hypertension.

Variables	OR (95% Cl)	*p* value
Age	1.060 (1.04–1.081)	<0.001
Sex	1.124 (1.032–1.443)	<0.001
Drinker	0.006 (1.110–1.150)	0.001
Smoker	1.178 (1.000–1.388)	<0.001
BMI	1.030 (1.030–1.126)	<0.001
Dyslipidemia	0.932 (0.886–1.063)	<0.001
Mean diameter of the left main renal artery	0.999 (0.996–1.001)	0.259
Mean diameter of the right main renal artery	1.006 (0.991–1.022)	0.425
ARA	0.748 (0.458–1.220)	0.245
MARB	1.033 (0.981–1.088)	0.214

Note: ARA: the accessory renal artery; MARB: main renal artery branches outside the renal hilum.

**Table 5:** Multivariate logistic regression for Diagnosed Hypertension.

Variables	Model 1		Model 2	
	OR (95% Cl)	*p* value	OR (95% Cl)	*p* value
ARA	0.954 (0.741–1.229)	0.718	2.069 (1.614–2.524)	0.002
MARB	1.095 (0.794–1.511)	0.579	2.071 (1.508–2.634)	0.001

Model 1: adjusted for age, sex, drinker, smoker, BMI, dyslipidemia., ARA, MARB. Model 2 included all variables in model 1 plus adjustments for mean diameter of left main renal artery and right main renal artery, CI confidence interval; HR hazard ratio.

## Data Availability

The datasets generated and analyzed during the current study are available from the corresponding author on reasonable request.

## References

[1] Murray C. J. L., Lopez A. D. (2017). Measuring global health: motivation and evolution of the global burden of disease study. *The Lancet*.

[2] Zeller T. (2015). Renal artery stenosis--new insights and developments. *Deutsche Medizinische Wochenschrift (1946)*.

[3] Chrysochou C., Kalra P. A. (2009). Epidemiology and natural history of atherosclerotic renovascular disease. *Progress in Cardiovascular Diseases*.

[4] Aboyans V., Desormais I., Magne J., Morange G., Mohty D., Lacroix P. (2017). Renal artery stenosis in patients with peripheral artery disease: prevalence, risk factors and long-term prognosis. *European Journal of Vascular and Endovascular Surgery*.

[5] Zheng B., Ma Q., Zheng L.-H., Yong Q., He Y.-H., Liu J.-H. (2015). Analysis of renal artery stenosis in patients with heart failure. *Chinese Medical Journal*.

[6] Amighi J., Schlager O., Haumer M. (2009). Renal artery stenosis predicts adverse cardiovascular and renal outcome in patients with peripheral artery disease. *European Journal of Clinical Investigation*.

[7] Mui K.-W., Zeebregts C. J., van den Hout H., van Baal J. G., Navis G., Jan-Woittiez A. (2011). Impact of incidental renal artery stenosis on long-term mortality in patients with peripheral arterial disease undergoing vascular procedure. *Journal of Vascular Surgery*.

[8] Cai G., Zheng Y., Sun X., Chen X., Survey of Prevalence, Awareness, and Treatment Rates in Chronic Kidney Disease Patients with Hypertension in China Collaborative Group (2013). Prevalence, awareness, treatment, and control of hypertension in elderly adults with chronic kidney disease: results from the survey of prevalence, awareness, and treatment rates in chronic kidney disease patients with hypertension in China. *Journal of the American Geriatrics Society*.

[9] Fengler K., Ewen S., Höllriegel R. (2017). Blood pressure response to main renal artery and combined main renal artery plus branch renal denervation in patients with resistant hypertension. *Journal of the American Heart Association*.

[10] VonAchen P., Hamann J., Houghland T. (2016). Accessory renal arteries: prevalence in resistant hypertension and an important role in nonresponse to radiofrequency renal denervation. *Cardiovascular Revascularization Medicine*.

[11] Mahfoud F., Edelman E. R., Böhm M. (2014). Catheter-Based renal denervation is No simple matter. *Journal of the American College of Cardiology*.

[12] Atas H., Durmus E., Sunbul M., Mutlu B. (2014). Successful accessory renal artery denervation in a patient with resistant hypertension. *Heart Views: the Official Journal of the Gulf Heart Association*.

[13] Ewen S., Ukena C., Lüscher T. F. (2016). Anatomical and procedural determinants of catheter-based renal denervation. *Cardiovascular Revascularization Medicine*.

[14] Palmieri B. J., Petroianu A., Silva L. C., Andrade L. M., Alberti L. R. (2011). Estudo do padrão arterial de 200 pedículos renais por meio de angiotomografias. *Revista do Colégio Brasileiro de Cirurgiões*.

[15] Schönherr R., Klinge M., Rudolph J. M. (2015). Real-time investigation of dynamic protein crystallization in living cells. *Structural Dynamics*.

[16] Satyapal K. S., Haffejee A. A., Singh B., Ramsaroop L., Robbs J. V., Kalideen J. M. (2001). Additional renal arteries incidence and morphometry. *Surgical and Radiologic Anatomy*.

[17] Zorgdrager M., Krikke C., Hofker S. H., Leuvenink H. G. D., Pol R. A. (2016). Multiple renal arteries in kidney transplantation: a systematic review and meta-analysis. *Annals of Transplantation*.

[18] Shakeri A. B., Shane Tubbs R., Shoja M. M. (2007). Bipolar supernumerary renal artery. *Surgical and Radiologic Anatomy*.

[19] Bakker J., Beek F. J., Beutler J. J. (1998). Renal artery stenosis and accessory renal arteries: accuracy of detection and visualization with gadolinium-enhanced breath-hold MR angiography. *Radiology*.

[20] Glodny B., Cromme S., Wörtler K., Winde G. (2001). A possible explanation for the frequent concomitance of arterial hypertension and multiple renal arteries. *Medical Hypotheses*.

[21] Verloop W. L., Vink E. E., Spiering W. (2014). Renal denervation in multiple renal arteries. *European Journal of Clinical Investigation*.

[22] Nemoto N., Hamann J., Lesser J., Schwartz R. (2014). Renal denervation in patients with accessory renal arteries: renal mass is directly proportional to total renal artery cross sectional area and implications for therapy. *Journal of the American College of Cardiology*.

[23] Gupta A., Tello R. (2004). Accessory renal arteries are not related to hypertension risk:A review of MR angiography data. *American Journal of Roentgenology*.

